# Characterisation of Cognitive Load Using Machine Learning Classifiers of Electroencephalogram Data

**DOI:** 10.3390/s23208528

**Published:** 2023-10-17

**Authors:** Qi Wang, Daniel Smythe, Jun Cao, Zhilin Hu, Karl J. Proctor, Andrew P. Owens, Yifan Zhao

**Affiliations:** 1 School of Aerospace, Transport and Manufacturing, Cranfield University, Cranfield MK43 0AL, UK; qi.wang.090@cranfield.ac.uk (Q.W.); daniel.smythe.685@cranfield.ac.uk (D.S.); jun.cao@cranfield.ac.uk (J.C.); zhilin.hu.729@cranfield.ac.uk (Z.H.); 2 Jaguar Land Rover Research, Coventry CV4 7AL, UK; kprocto1@jaguarlandrover.com (K.J.P.); aowens5@jaguarlandrover.com (A.P.O.)

**Keywords:** electroencephalography, machine learning, Deep Neural Network, Support Vector Machine, cognitive load classification

## Abstract

A high cognitive load can overload a person, potentially resulting in catastrophic accidents. It is therefore important to ensure the level of cognitive load associated with safety-critical tasks (such as driving a vehicle) remains manageable for drivers, enabling them to respond appropriately to changes in the driving environment. Although electroencephalography (EEG) has attracted significant interest in cognitive load research, few studies have used EEG to investigate cognitive load in the context of driving. This paper presents a feasibility study on the simulation of various levels of cognitive load through designing and implementing four driving tasks. We employ machine learning-based classification techniques using EEG recordings to differentiate driving conditions. An EEG dataset containing these four driving tasks from a group of 20 participants was collected to investigate whether EEG can be used as an indicator of changes in cognitive load. The collected dataset was used to train four Deep Neural Networks and four Support Vector Machine classification models. The results showed that the best model achieved a classification accuracy of 90.37%, utilising statistical features from multiple frequency bands in 24 EEG channels. Furthermore, the Gamma and Beta bands achieved higher classification accuracy than the Alpha and Theta bands during the analysis. The outcomes of this study have the potential to enhance the Human–Machine Interface of vehicles, contributing to improved safety.

## 1. Introduction

Cognitive load refers to the amount of working memory required to complete a task within a specified time. Driving tasks require drivers to allocate specific amounts of physical and cognitive loads. Cognitive load plays a vital role in daily driving activities. The Department of Transport of the UK government issued a Reported Road Casualties Great Britain Annual Report in 2021 [1], showing that approximately 22% of road accidents caused by distraction were serious or fatal. Numerous distractions, such as performing secondary tasks, roadside distractions, and talking, can add to a driver’s cognitive load. Increased driving-related cognitive load can impact the driver’s attention and lead to fatigue [2]. Measuring drivers’ cognitive loads is important for improving the understanding of driver behaviours and is thus worth investigating to enhance driver–vehicle interactions. Considerable studies on measuring and estimating drivers’ cognitive loads have been conducted using various methodologies in recent years. These methods fit into three different categories [3]:

- Subjective Measures are conventionally measured with a questionnaire, asking subjects to rate a task’s difficulty. The NASA Task Load Index (TLX) [4,5,6,7,8] and Subjective Workload Assessment Technique (SWAT) [9] are the two most common subjective measurement methods used in this category. Subjective measurement cannot evaluate time-varying qualities, and this method can also be affected by events towards the end of the experiment at the time of the questionnaire’s administration [5,6].
- Performance measures—the performance of a participant is evaluated to gain insight into cognitive load. Driving quality, response latency, and reaction times are frequently used to gauge performance. Such measures are also often employed in detecting distraction and fatigue. Various factors can influence performance measurement, including individual differences, participants’ skill levels, and external variables [10,11,12].
- Psychophysiological measures include brainwave activity, heart rate variability correlated with task demands [13], eye blinking, pupil diameters, and head rotational angles [14].

Electroencephalography (EEG), which records brain electrical activity, is a highly attractive research method in driver-related cognitive load research. Signals from the brain are propagated through the skull and detected on the scalp [15]. Due to the scalp’s relatively low conductivity, EEG devices typically detect signals originating from the brain’s cortices. EEG devices offer high temporal resolution (>1 kHz) but have limited spatial resolution, typically ranging from 5 to 9 cm [16]. These signals tend to be noisy, with a low signal-to-noise ratio due to susceptibility to physiological phenomena, such as muscle movements, resulting in contaminated EEG data. The most common artefact originates from eye blinks. The features used to classify EEG data in driving-related research vary significantly across the literature. Some studies use frequency bands, such as Delta (0.5–4 Hz), Theta (4–8 Hz), Alpha (8–14 Hz), Beta (14–30 Hz), and Gamma (30–100 Hz), although the specific frequency ranges chosen may differ [17,18,19,20,21,22,23,24,25,26]. Other studies use features generated from Power Spectrum Density (PSD) [27,28]. In addition to these frequency-based features, features in the time and spatial domains have been well-studied [28,29,30]. Furthermore, a wide variety of classification models have been reported to further group these features. The most common methods include Support Vector Machines (SVMs), Long Short-Term Memory (LSTM) [17,31,32,33], Artificial Neural Networks (ANNs) [19], Stacked Denoising Auto-Encoders (SDAEs) [25,27], and Convolutional Neural Networks (CNNs) [17,18,19,24,30,33,34,35,36].

In recent years, there has been a growing interest in Functional Near-Infrared Spectroscopy (fNIRS) as a research tool. It measures changes in cerebral blood flow (CBF) and related haemoglobin concentrations by utilising near-infrared light sources and detectors placed on the scalp. fNIRS offers portability similar to EEG while avoiding electromyographic and blink artefacts. However, it has fewer channels compared with EEG recordings and can be susceptible to variations in skin perfusion and scalp blood flow [16]. Aghajani et al. [8] achieved an accuracy ranging from 68% to 87% in classifying different cognitive levels during N-Back tasks using an SVM model. Compared with recent fNIRS research [37,38,39], there has been limited research utilising EEG to classify cognitive load whilst driving. Notably, the study completed by Almogbel et al. [34] was based on the results of a single participant, achieving an accuracy of 87% by using SVM with a Convolutional Neural Network autoencoder (CNN-AE) as a feature extraction method for EEG classification. Cui et al. [11] classified engagement, enjoyment, boredom, and frustration where the cognitive load is not the main focus. It should be noted that in most existing studies, cognitive load levels are simulated using various non-driving tasks, typically N-Back tasks [40], or other visual or auditory tasks, such as the image-responding tasks in research by Takada et al. [41] or the auditory stimuli and calculation task in research by Faure et al. [42]. These tasks often demand high-level attention and can exhibit different workload levels relatively easily, but they lack the context of driving, which requires middle-level attention.

This research proposes a method for classifying different driving environments and traffic densities that demand different cognitive load levels, using various DNN and SVM machine learning-based classifiers. Unlike existing studies that focus on secondary tasks, the novelty of this paper lies in characterising the driver’s cognitive load effects during various driving tasks, utilising EEG recordings collected from experimental studies. The main contributions of this study include:

- A simulator-based experiment to generate different levels of cognitive load based on four carefully designed driving tasks.
- Extraction of frequency-based features used to classify driving conditions by employing DNN and SVM machine learning-based classifiers.
- An exploration of the topological patterns of different frequency bands of brain waves under driving tasks demanding varying levels of cognitive load.

## 2. Methodology

**Figure 1:**
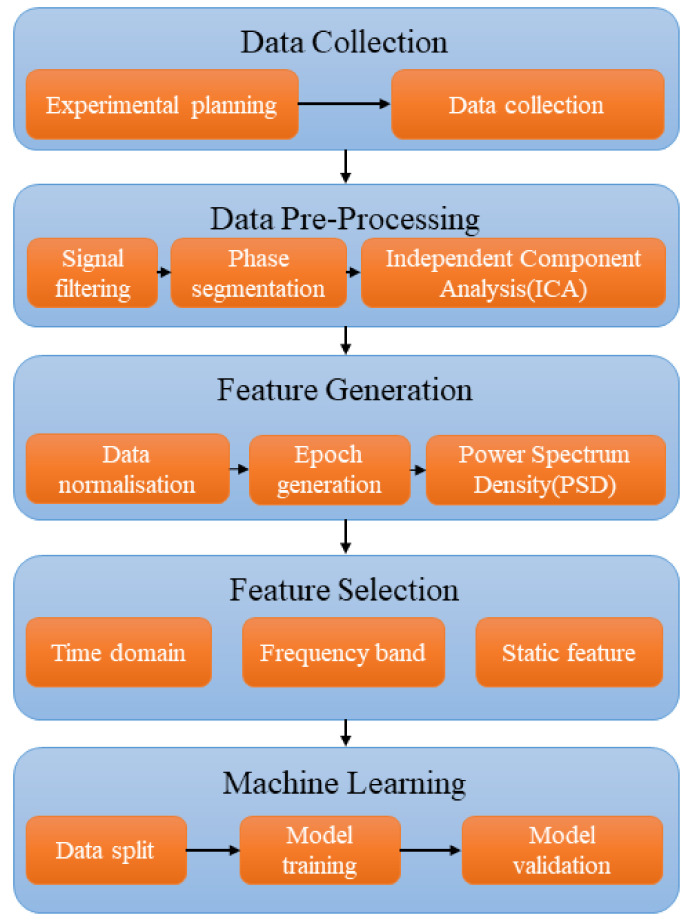
A block diagram displaying the four steps of the overall methodology.

The methodology of this study consists of four main segments: data collection, data pre-processing, feature generation, feature selection, and machine learning, as shown in Figure 1. The data collection stage explains the experimental setup and the data collection goals. The data pre-processing section describes how the obtained data are processed to improve quality and remove artefacts due to eye movements. Feature generation describes how EEG-based features are extracted from the dataset and prepared for machine learning. Feature selection tests different features in frequency and time domains across different EEG channels. Finally, machine learning describes the classification of datasets of features using chosen machine learning models.

### 2.1. Data Collection

**Figure 2:**
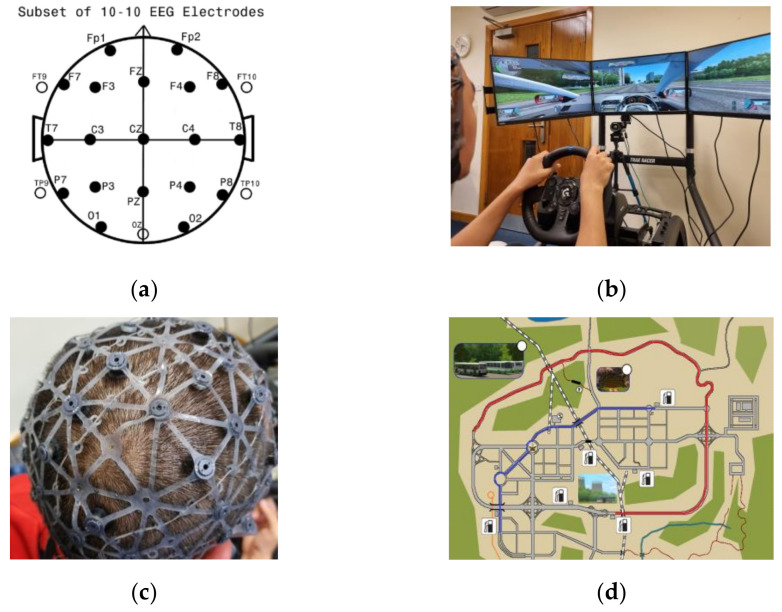
Images displaying the experimental setup and a map showing routes followed by participants. (**a**) Complete sets of sensors and their locations. (**b**) Driver simulator setup. (**c**) Display of the EEG sensor device. (**d**) Route map of the experiment; the red route shows the motorway route, and the blue route shows the urban route.

EEG data were collected for this experiment using a water-based Waveguard Net from Ant Neuro. The device has 24 electrodes and is referenced from the Cz electrode, positioned according to the international 10–20 system [43]. The sample rate was 256 Hz. The device is relatively simple to set up compared with gel-based EEG caps, but it must be bathed in saltwater for at least 10 min before fitting and recording. The complete set of sensors and their locations are shown in Figure 2a. The device is shown in Figure 2c, and one snapshot of the whole experiment setup is shown in Figure 2b. A total of 20 male participants took part in the experiment. Each participant gave informed consent for this research to use and publish the aggregated data. The average age of participants was 27.65 years old (ranging from 22 to 42), and they all owned valid driving licenses. In addition, three participants consumed coffee within 12 h of the experiment. Most of the experiments were conducted in the morning, and participants were encouraged to ensure they had adequate sleep, which was verified through the questionnaire. Experiments were conducted in a quiet office area with stable lighting and air conditioning to minimise external interferences that cause additional eye blinks or head movement. Throughout experiments, participants were not allowed to engage in conversation with others.

The City Car Driving software was used for driving simulation. City Car Driving is a PC platform driving simulation software designed for car learners to practice their driving skills. This software can simulate different driving environments with different intensities of cars and pedestrians. All participants were required to drive in automatic gear, partially for simplicity and partly to limit the amount of muscle movement (to reduce the amount of noise in EEG data). There were five sessions in the experiment:

- Practice: Participants were given time to become familiar with the driving simulator and simulation software. After eight minutes, they were prompted and asked whether they wanted to start the experiment. They could become familiar with the system for as long as they wanted.
- Motorway—no cars: Participants were asked to follow a motorway route until being prompted to pull over on the left. In this session, there were no other cars (mean time taken: 6 min and 57 s).
- Motorway—with cars: Participants repeated the same route as above. However, this section included AI-controlled cars on the road (vehicle traffic density of 70%; mean time taken: 8 min and 36 s).
- Urban—no cars: Participants were asked to travel along a route in an urban environment and follow instructions given by a GPS included in the software. In this session, there were no other cars (mean time taken: 6 min and 1 s).
- Urban—with cars: Participants repeated the same route as above. However, this section included AI-controlled cars on the road (vehicle traffic density of 30%; mean time taken: 8 min and 6 s).

Each session took approximately 7–10 min and all participants were given a 5 min break between each section to reduce the effect of build-up fatigue. Figure 2d illustrates the motorway route (shown in red) and the urban route (shown in blue). The motorway section consists of approximately 5 km of a single carriageway and approximately 8 km of a motorway. The speed limit for driving on the motorway section is 110 km/h and 90 km/h on the single carriageway section. The urban route comprises a combination of dual and single street roads, with a total length of approximately 8 km. The urban route includes three traffic light junctions and two roundabout junctions, and all participants are required to follow UK driving regulations at these junctions. In addition, the protocol was counterbalanced so that half of the participants performed two motorway routes first, while the other half completed urban routes first. This arrangement aimed to ensure that any apparent decrease in cognitive load was not due to the participant feeling more familiar with the simulation.

**Figure 3:**
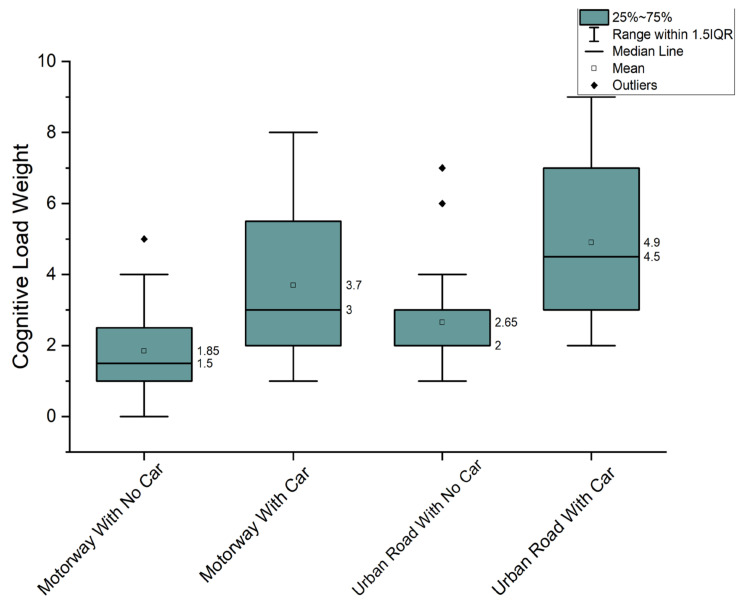
The cognitive load of driving tasks rated by participants.

After completing all sessions, participants were required to rate the cognitive load of four driving tasks (the training session was excluded) on a scale ranging from 1 to 10, with 1 indicating the lowest cognitive load level and 10 representing the highest cognitive load level. The outcomes are summarised in Figure 3. As expected, “Motorway—no car” was rated as the task with the lowest load (mean: 1.9); “Urban—with cars” was rated as the task with the highest load (mean: 4.9). Statistical analysis using ANOVA was applied to the questionnaire results. The *p*-values for driving scenarios with cars and driving scenarios without cars were found to be 0.00112 for the motorway route and 0.00053 for the urban route. This indicates that the cognitive load during driving tasks with other cars is significantly higher than that without other cars. Therefore, this experiment design is promising to potentially simulate scenarios that require high or low cognitive loads.

Additionally, the rated load of the driving tasks with other cars exhibits greater variation than that without other cars. Furthermore, these four tasks potentially represent four different levels of cognitive load. This research hypothesises that the classification of levels of cognitive load can be represented by the classification of EEG data of participants completing these driving tasks.

### 2.2. Data Pre-Processing

Data from the beginning stage of each session and durations when the driving task was interrupted by accidents or misoperation were excluded. These periods cannot accurately reflect the desired driving workload. In total, 47,000 s of data were collected for all participants, of which 35,000 s were preserved for analysis. This study employed the MNE software package (an open-source Python package for exploring, visualising, and analysing human neurophysiological data) [44] in Python to process the collected EEG data. Firstly, a band-pass filter [0.2 Hz, 40 Hz] was applied to all data. The filter was applied using a Hamming window with a 0.0194 Hz passband ripple and a 53 dB stopband attenuation. Additionally, a low-pass frequency of 40 Hz was chosen to remove noise from the power source. The EEG data from the entire experiment was separated into four segments based on four driving tasks, while the data in the practice phase were neglected. To reduce the interference of muscle activities, such as eye blinks, we applied Independent Component Analysis (ICA). It blindly decomposes a signal into a collection of signals from differing sources using the fastica method in the ‘MNE’ module with 18 principal components. ICA can determine which parts of EEG signals constitute artefacts due to the movements of ocular muscles. The decomposed signals must be manually labelled, as those are believed to represent the ocular artefacts. Since the ICA cannot perfectly identify the ocular artefacts and there will be some human errors, the artefacts can never be removed entirely but are significantly reduced using this method.

### 2.3. Feature Generation and Feature Selection

This step comprises two subsections: feature generation, which primarily focuses on noise reduction and creating features for subsequent selection, and feature selection, which outlines the methodology for choosing various features for building the subsequent model.

#### 2.3.1. Normalisation and Dataset Generation

Before generating the dataset for machine learning-based classification, the EEG data were standardised for each channel to ensure the EEG signals from each participant were normalised with a mean of 0 and a variance of 1. This operation was applied to each segmented data portion before generating a set of features.

Before learning about features, the data were split into discrete time intervals to increase the number of instances. For this purpose, the data were segmented into 1 s time intervals using a sliding window with a 50% overlap. Before training the model, another round of pre-processing was applied for each segment to further reduce the impact of noise. We used a threshold equal to the 95th percentile of the Peak-to-Peak (PTP) values across the data entries individually in each class. It removes any data segment containing a channel with a PTP value above that threshold. PTP is the distance from the minimum to maximum peak representing each data entry’s signal range. Any entry with a channel containing a PTP value above the threshold is deemed too noisy and removed from the dataset on which the model was trained and validated. After PTP removal, we were left with 11,559 segments for the session of motorways without cars, 15,211 segments for motorways with cars, 12,131 segments for urban without cars, and 14,760 segments for urban with cars.

#### 2.3.2. Feature Selection

It is evident that the PSD of EEG recordings can effectively measure workload [45], and, therefore, it was used as a single feature in this study. Four frequency bands are defined as Theta (4–8 Hz), Alpha (8–14 Hz), Beta (14–30 Hz), and Gamma (30–40 Hz). First, the PSD using the Welch method [46] was generated for each data entry to produce these features. For each data entry, a PSD was generated using 72 frequency values ranging from 4 to 40 Hz with a resolution of 0.5 Hz.

The selected statistical features for each band include the mean, variance, and absolute maximum amplitude of frequency response. In addition, the band power was generated by integrating the PSD between selected frequency ranges, which was then divided by the total power of the entire frequency range. This step gives a fraction representing the ratio of power this frequency band contains with respect to the full frequency range. Each feature was generated for each EEG channel and each frequency band, giving a set of 4 × 4 × 24 = 384 total features.

### 2.4. Machine Learning

For this study, the selected features were classified using a Deep Neural Network (DNN) and a Support Vector Machine (SVM). The ‘Keras’ Module in the Tensorflow Python library was used [47] to implement DNN, and the ‘Sklearn’ library was used to implement SVM [48].

The first layer of a DNN is the input layer, which contains an array of input values used as the feature inputs to the network. Next are fully connected layers, where the main computation of the model takes place. Each edge in the diagram has an associated weight value. The nodes in the current layer are multiplied by the weight along the edge connected to the node to produce values associated with each node in the next layer. In a fully connected network, each node is connected to each node in the next layer. Once the value is obtained for the node, the next layer of nodes can be computed. An activation function is applied to each layer’s output to augment the results. After calculating all the fully connected layers, the output layer can be computed using the same method. An activation function is applied to the output values, and a set of values associated with each class type is obtained. Then, the most probable class is taken as the output class. The training phase consists of learning the values associated with each edge by continuously passing a set of data through the network and updating the edges according to the error in the output. The exact method to update the edge weights depends on the optimiser in ‘Keras’ [49].

Eight different models were trained, half with a DNN and the other half with an SVM. There are four classification tasks:

- Motorway-High-Low: The condition with other vehicles on the road was labelled as high intensity of cars (indicating high cognitive load scenarios), and the condition without other road users was labelled as intensity of cars (indicating low cognitive load scenarios). Only the motorway data are included in this task.
- Urban-High-Low: The labelling process is the same as Motorway-High-Low, but only the urban data are included.
- Combined-High-Low: The labelling process is the same as Motorway-High-Low, but the urban and motorway data are included.
- Four-Class: In this task, each experiment session is given a separate class, resulting in four classes: urban with no cars, urban with cars, motorway with no cars, and motorway with cars.

**Figure 4:**
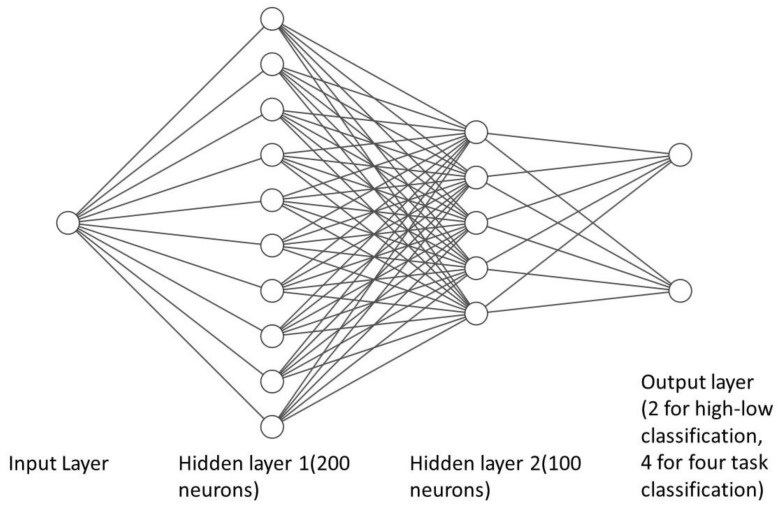
The architecture of the DNN model.

Figure 4 shows the architecture of the used DNN model. The DNN architecture includes two layers: one with 200 fully connected neurons and another with 100 fully connected neurons. In three high-low tasks, there are two classes and, therefore, two output layers, while in the four-class task, there are four classes and, therefore, four output layers. All output layers used a sigmoid activation function for all tasks. In addition, a binary-cross-entropy loss function and the Keras’ Adam’ optimiser [50] were used for both 2-class and 4-class classification tasks. The first layer used a linear activation function, and the second used a Relu activation function. An epoch size of 200 was used to train the model. In addition, three different measurements were used to represent the performance of the produced models. The first is accuracy, which is represented by the following formula:

(1) $$Accuracy=\frac{TP+TN}{TP+FP+FN+TN}$$

where *TP* represents the True Positive, *TN* is the True Negative, *FN* is the False Negative, and *FP* is the False Positive. This value represents the ratio of correct classifications to incorrect classifications. When dealing with multiple classes, this is averaged across all classes. However, additional performance values were measured since there may be a bias in accuracy due to an unbalanced dataset. The following formula represents precision:

(2) $$Precision=\frac{TP}{TP+FP}$$

The precision represents the number of correct classifications out of the total number of dataset entries identified as that class by the model. The following formula represents recall:

(3) $$Recall=\frac{TP}{TP+FN}$$

The recall represents the number of correct classifications from the total number of dataset entries in that class. In addition to these classification performance indicators, a confusion matrix is produced for each task to analyse the detailed performance.

For each model, average accuracy, precision, and recall were generated by training the model using 10-fold cross-validation and leave-one-out cross-validation. For the 10-fold cross-validation, the entire dataset was split into ten equally sized sets. Ten different datasets for machine learning were generated, using one of these folds as the testing set and the remainder as the training set. This step was repeated ten times to ensure each folder was used for validation. The drawback of using only one testing set is that the test accuracy can vary greatly depending on which observations were used in the training and testing sets. The resulting accuracy could be much larger or smaller if we used a different set of observations for the training and testing sets. The k-folder cross-validation was employed to address this limitation.

The leave-one-person-out cross-validation applies the same concept, but the data are divided into different folders based on participants. The data from one participant are used as the testing set, and others are used for training. This step is repeated 20 times in this study to ensure each participant is used for validation. This validation approach aims to test the performance of a model that learned from a group of subjects testing on another subject to measure the variation across subjects.

## 3. Results

**Figure 5:**
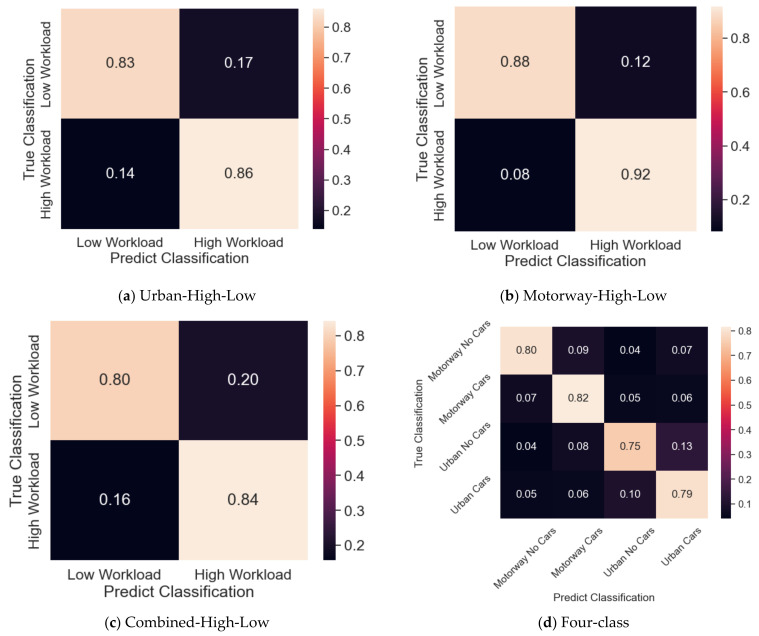
Classification matrices using DNN for 10-folder cross-validation. (**a**) Classification matrices of High-Low two-class classification in urban environments. (**b**) Classification matrices of High-Low two-class classification in motorway environments. (**c**) Classification matrices of High-Low two-class classification combined. (**d**) Classification matrices of four-class classification.

Table 1 shows the model performance of two validation approaches for four classification tasks using DNNs and SVMs. Using the selected features from EEG recordings, the DNNs can effectively distinguish high and low intensities of cars with an accuracy of around 85%. Even the four-class classification achieved an accuracy of up to 78%, which indicates the different brain activities during the four driving tasks. Results also suggest that when using the 10-fold cross-validation, the DNNs entirely outperform the SVMs. The urban data show an 18.21% difference between the two models, and the motorway data show a 17.98% difference. It is also observed that the two models behave similarly for the Combined-high-low task and four-class, increasing by 17.41% and 29.90%, respectively. Additionally, the accuracy, precision, and recall values are very close in each model, meaning the models are not heavily biased towards one class in particular. Also, the classification matrices of the DNN presented in Figure 5 show no extreme bias towards one class when using the 10-fold cross-validation.

However, the same trend is not observed when analysing results from the leave-one-person-out validation. The results from both methods in Table 1 are close to the baseline of 50% for the first three classification tasks and 30% for the last task. It suggests that the features’ pattern that can distinguish high and low intensities of cars differs across participants. The model performs more accurately when it sees data from each individual during training. The 10-fold cross-validation mixes each individual’s data between the training and validation sets. During the validation process, there is a high chance that the model sees this individual’s data previously but in different epochs and learns key features from them. There is, however, a chance that most of these learnt features are individually dependent. The algorithm behaves similarly to a random method for features learnt from certain subjects and tested on other subjects. This observation implies that the learned features are individually specific. However, this can still be useful to determine the load level for individuals where specific machine learning model learns.

The results shown here may link back to prior research about how cognitive load is defined in the literature review. As suggested by O’Donnell and Eggemeier [50], the cognitive load is “The portion of an individual’s limited mental capacity that is required by task demands”. Cognitive load depends on working memory [51], and individuals’ perception and cognitive functions to complete a specific task can differ based on their experiences and familiarity. Some individuals may have found each task in this experiment extremely difficult and experienced a much higher load in the same tasks that others found simple.

It was found that the results displayed by using the 10-fold cross-validation are comparable to results shown by other experiments. For example, So et al. [21] reported results ranging from 60.40 to 76.00% using an SVM to classify two levels of cognitive load, while the results in this study display a range of 64.24–72.39% accuracy using an SVM with two classes. However, it should be noted that very limited studies presented the results from a cross-participant validation. Cui et al. [11] used a cross-participant validation and found large variations in some results. The proposed relative power + SVM model had an accuracy ranging from 30.77 to 89.84% between subjects, suggesting the difficulty of finding consistent features to differentiate cognitive load levels for all subjects.

Although the accuracy drops as expected for the four-class model, EEG data can separate these four tasks efficiently with more than 77% accuracy. There may be a variation in the cognitive load resulting in a change in feature values due to the GPS, turns, and roundabouts in the urban route. In other words, the cognitive load level for each task can be different. The model does suffer the same issue as the other three models with cross-participant validation, obtaining an accuracy within 1σ of the random accuracy of 25%.

## 4. Discussion

This section discusses and analyses the results to uncover significant findings. In the first subsection, various other features, including power ratio, mean, variance, and maximum values, are examined and compared with PSD. The frequency band analysis subsection involves assessing different frequency bands, accompanied by the generation of a topographic map. The limitations of the study are addressed in the last subsection, discussing various constraints encountered.

### 4.1. Feature Analysis

This subsection explores the contribution to the classification of each feature. A dedicated model was trained for each feature type based on bands and statistical values. There are 32 models for four bands and four statistical values, and they were performed for two-class datasets. The four-class dataset is not investigated here, as this analysis aims to look at each feature’s impact on classifying the cognitive load. As analysed previously, the cognitive load distinction is more apparent in two-class datasets. In addition, a DNN with fewer neurons for each feature is available for the four-class dataset. The models were trained using two layers of 10 neurons to reduce the amount of potential overfitting that might occur.

Table 2 displays the results for the four bands and four statistical values. It can be observed that the accuracy is higher when using higher frequency bands. The true reason for this observation is unclear. However, there are studies suggesting that the Beta wave is a carrier of visual attention in humans [51], and Gamma waves are shown to increase when given a visual stimulus [52]. Both factors may cause this observation, as there is an increase in visual stimuli when other cars are included on the road. These frequency bands are further analysed in a later section.

In addition, whilst a high performance is observed in each statistical feature, the variance measure tends to perform most accurately in each dataset. In the Motorway-High-Low dataset, the variance feature achieved an accuracy of 79.36%, which was 8.56% greater than the following highest result. In the Urban-High-Low dataset, it performed 3.34% better; in the Combined-High-Low dataset, it performed 3.49% better. Among the remaining features, no metric performs significantly worse than the rest. The differences observed are probably due to random chance, as they remain within either 1σ or 2σ of each other.

**Figure 6:**
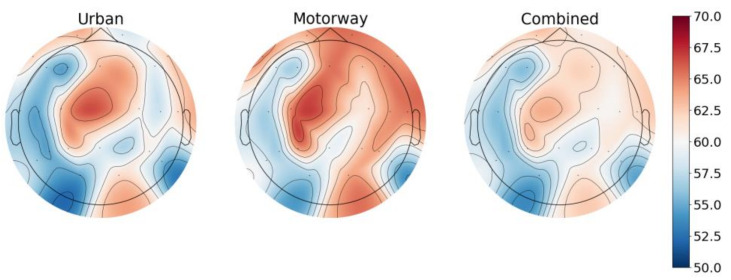
Topographic maps of the accuracy values in each model.

Figure 6 shows the results of the channel analysis displayed on a topographic map. There is an apparent trend in the accuracy produced for each channel. In this diagram, the red region represents regions of higher accuracy, and the blue region represents regions of lower accuracy. The exact accuracy values are indicated on the colour bar. The greatest-performing regions are consistent throughout each dataset. The most accurate regions occur mainly along the frontal lobe, right temporal lobe, parietal lobe, and occipital lobe. Regions with the most prominent accuracy among channels include ‘Fp1’, ‘Fp2’, ‘Fz’, ‘F4’, ‘F8’, ‘FT10’, ‘C3’, ‘C4’, ‘T8’, ‘TP10’, ‘P3’, ‘Pz’ ‘P4’, ‘P8’, and ‘O2’. In addition, no channel performed worse than or within 2σ of 50%. Overall, the channel accuracy measurements ranged from 54.96 to 65.38%.

### 4.2. Frequency Band Analysis

An investigation of frequency bands was performed to observe whether there were any significant trends in the individual frequency bands across the experiments. This was mostly inspired by trends in the previous section, where the accuracy values increased as the frequency values of the bands increased. For this purpose, the mean and variance values of Theta, Alpha, Beta, and Gamma were plotted by taking the median response from each participant in each experimental segment. The purpose of taking the median was to ignore anomalous values where the orders of magnitude were greater than other values.

**Figure 7:**
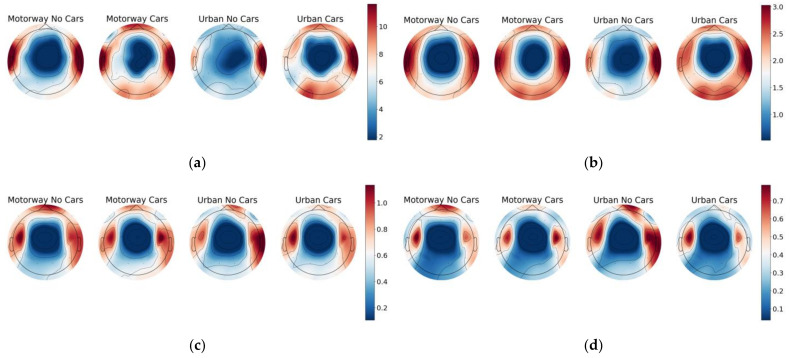
Topographic maps displaying median means of the Theta, Alpha, Beta, and Gamma bands across each section of experiment. (**a**) Theta band. (**b**) Alpha band. (**c**) Beta band. (**d**) Gamma band.

**Figure 8:**
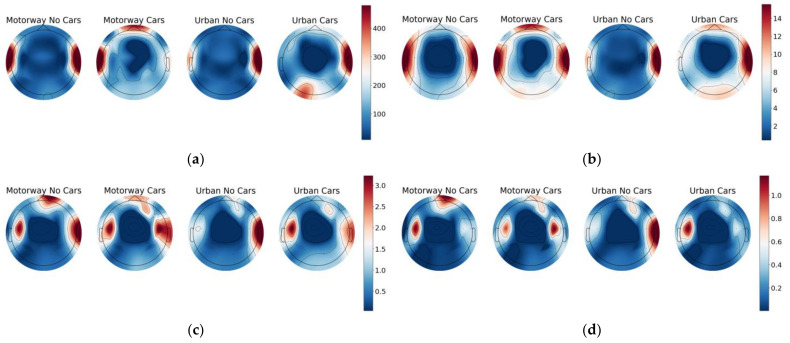
Topographic maps displaying the variance in Theta, Alpha, Beta, and Gamma bands across each experiment section. (**a**) Theta band. (**b**) Alpha band. (**c**) Beta band. (**d**) Gamma band.

Figure 7 and Figure 8 display the results of this investigation, where some very noticeable trends are observed. The mean and variance in each diagram’s middle parietal and occipital regions consistently exhibited the smallest response values, while the frontal and side regions showed significantly larger responses. Additionally, the contour shapes within each band are highly similar. However, they differ in the intensities displayed within each region, and there are noticeable differences between each classification task.

As should be expected, the intensity of the mean and variance decreased throughout each band due to the natural shape of the PSD expected from the EEG data. Finally, there are noticeable commonalities in shapes displayed by the distributions shown by the two classes with cars and the two classes without cars. Figure 7 shows that although every class peaks within the left and right temporal regions, both classes with cars display a reduced response at the frontal region compared with those without cars in the Beta and Gamma bands. In the Theta and Alpha bands, classes with cars show a stronger frontal response than those without cars. A similar trend is seen in Figure 8. However, such commonalities may be entirely down to random chance, and more work should be put into a detailed investigation of the specifics of the frequency band behaviours in future works.

### 4.3. Limitations

Although the goals set for this study were met, there are some limitations which need to be addressed in future studies. Firstly, in this experimental design, a simple self-designed questionnaire was used to rate the cognitive load of each driving task. This could lead to bias in rating the cognitive load with a single parameter. Conducting a NASA-TLX questionnaire after all four sessions is challenging for participants due to memory constraints. Essentially, the cognitive load levels simulated in this study are relative rather than absolute, and we recognise this as a potential limitation. During the experiment, we used an all-male cohort who we did not ask to refrain from neuroactive stimulants, such as caffeine and nicotine. The influence of pulse and lateral eye movement in EEG was considered during the analysis. Moreover, only the PSD was used as the feature for classification. In future studies, we plan to incorporate additional features, such as amplitude-based features, brain functional connectivity, and effective connectivity to improve classification performance further. Although the models perform well with the 10-folder cross-validation, they fail to perform at the same level using the cross-participant validation. It implies the information/pattern the models learned from the dataset is not general enough, and more subjects should be included in a more diverse (e.g., gender, age) and well-described (e.g., quality of previous night’s sleep) cohort of participants to enhance these positive initial findings.

## 5. Conclusions

Through a dedicated experiment, this study investigated drivers’ brain activity patterns measured with EEG techniques for four driving tasks that required different levels of cognitive load. Machine learning approaches were employed to classify the features extracted from EEG recordings. This aim can be deemed a success, as the developed models were able to classify the dataset between different tasks when using the 10-cross validation. In addition, the classification accuracy values achieved for high and low cognitive loads were comparable to the results of similar experiments, achieving accuracy values within the ranges of 81.65 to 90.37%% when using a DNN and 64.24 to 72.39% when using an SVM. Furthermore, the models could distinguish between all four classes with a 78.66% accuracy using a DNN and a 48.76% accuracy using an SVM.

A collection of small DNNs trained on small samples of features could be used to estimate each feature’s contribution. EEG channels in the scalp’s frontal, central, and right regions were found to perform noticeably better. In addition, the Gamma and Beta bands performed significantly better than the Theta and Alpha bands. This observation supports the findings of the existing literature, which implies that Gamma and Beta bands tend to change in response to visual stimuli and tasks [46,47]. Furthermore, it was found that the variance measured outperformed other statistical measures for the PSD regions by between 3.34 and 8.56%, depending on the utilised dataset.

## Figures and Tables

**Table 1:** Performance of the DNN and SVM classifiers on four data sets, using 10-fold cross-validation and cross-participant validation.

Method	Classification Task	10-Fold Cross Validation			Cross-Participant Validation		
		Accuracy	Precision	Recall	Accuracy	Precision	Recall
DNN	Urban-High-Low	84.26 ± 0.75%	84.27 ± 0.75%	84.26 ± 0.75%	49.52 ± 9.40%	49.53 ± 9.37%	49.54 ± 9.36%
	Motorway-High-Low	90.37 ± 0.55%	90.37 ± 0.57%	90.37 ± 0.59%	59.52 ± 13.37%	59.54 ± 13.35%	59.52 ± 13.36%
	Combined-High-Low	81.65 ± 0.73%	81.64 ± 0.73%	81.65 ± 0.72%	56.28 ± 6.41%	56.28 ± 6.41%	56.28 ± 6.41%
	Four-Class	78.66 ± 0.56%	77.61 ± 0.73%	76.70 ± 0.94%	31.39 ± 6.76%	31.91 ± 7.24%	28.59 ± 6.50%
SVM	Urban-High-Low	66.05 ± 1.15%	65.87 ± 1.10%	66.00 ± 1.08%	48.96 ± 9.98%	48.42 ± 10.53%	48.65 ± 9.31%
	Motorway-High-Low	72.39 ± 0.74%	72.72 ± 0.77%	73.18 ± 0.73%	58.26 ± 11.91%	58.79 ± 12.94%	57.46 ± 11.95%
	Combined-High-Low	64.24 ± 0.53%	64.65 ± 0.54%	64.83 ± 0.55%	55.54 ± 7.66%	56.63 ± 7.94%	55.40 ± 6.96%
	Four-Class	48.76 ± 0.66%	48.79 ± 0.70%4	49.00 ± 0.75%	30.47 ± 7.08%	30.77 ± 8.12%	30.03 ± 7.02%

**Table 2:** Accuracies of the band and statistical features of each dataset.

Feature	Urban-High-Low	Motorway-High-Low	Combined-High-Low
Theta Band	64.22 ± 0.62%	67.66 ± 1.18%	62.99 ± 0.61%
Alpha Band	67.04 ± 0.77%	70.46 ± 0.70%	64.97 ± 0.59%
Beta Band	75.23 ± 0.69%	80.46 ± 0.87%	72.26 ± 0.86%
Gamma Band	81.04 ± 1.46%	87.74 ± 0.75%	79.33 ± 0.71%
Power Ratio	69.26 ± 1.41%	69.10 ± 0.89%	67.01 ± 0.93%
Mean	68.79 ± 0.96%	70.80 ± 1.20%	66.96 ± 0.78%
Variance	72.69 ± 0.91%	79.36 ± 1.06%	70.50 ± 0.45%
Absolute Max	66.63 ± 0.80%	70.02 ± 1.00%	65.25 ± 0.66%

## Data Availability

The data presented in this study can be downloaded from: https://doi.org/10.17862/cranfield.rd.23978112 (accessed on 4 September 2023).
